# Supplementary material for: ‘Integrating Ethics and Equity with Economics and Effectiveness for newborn screening in the genomic age: A qualitative study protocol of stakeholder perspectives
Source: PLoS One. 2024 Mar 25;19(3):e0299336. doi: 10.1371/journal.pone.0299336 (PMC10962853; doi:10.1371/journal.pone.0299336)
Supplement: S1 Appendix — (DOCX) [file pone.0299336.s001.docx]

**S1 Appendix**

**gEnomics4newborns: In-depth interviews with consumers, health professionals, scientists, and policy makers**

**Interview Discussion Guide – Parent/Carer**

**General information:**

Ask if the participant would require an interpreter to participate in the interview. Introduce yourself and the purpose of the research. Newborn screening (NBS) is a way of screening to detect individuals for whom early diagnosis and treatment of ‘actionable’ conditions offers health benefits primarily to the individual. Currently, Australia screens for around 50 conditions and there is 98% public confidence in the program. Genetic screening tests look for changes (sometimes called variations in your DNA). DNA is packaged in genes, and these carry the instructions for all of your body functions. Sometimes, changes within your DNA may cause or put you at higher risk of an illness or condition and genetic screening helps us identify who is at more risk of a certain condition. There is now potential to add genetic screening tests (either single gene tests looking at changes in one gene, panels of genetic tests looking at changes in several genes, or screening for changes in the whole genetic code) for the first time to NBS, allowing us to potentially diagnose many more of these conditions in the newborn period. However, there are potential benefits and harms of this new technology and we would like to explore these ideas with you as you have received a genetic diagnosis for your child. The purpose of this research is to explore parents understanding of genomic NBS, your expectations of this process, and supports and information required to make informed decision about participating in future genomic NBS programs. We will use this information to help us understand what is important for parents in genomic NBS and how we may improve the health benefits, reduce the risks of harms to families and provide a model which is equitable, fair and ethical to all Australian families.

1. Ask if participant is happy to have to conversation recorded?
2. There are no right or wrong answers, feel free to provide as much or as little information as you like.
3. If you feel like you need a break, please feel free to stop at any time.
4. Even though we are taping our conversation, nothing you say will be linked back to you personally. The information we gather today will be combined with other interviews and reported as being anonymous.

- **Tell me about your experiences of your child’s condition**
  - What condition do they have?
  - How old were they when they were diagnosed?
  - What is your child’s current level of function? (*prompts* Day-to-day and/or overall function. Neurological and/or physical function)
  - What was your journey like to achieve a diagnosis? (*prompt* NBS vs clinical symptoms vs family history of genetic condition)
  - How has the diagnosis impacted you and your family? (*prompt* including the way and timing that your child was diagnosed)
  - How is your child’s care managed? (*prompt:* clinically? And day-to-day?)
  - What has worked well for you and your family in the way your child’s genetic condition has been managed?
  - Are there any challenges in managing your child’s genetic condition?
  - What are the emotions you experience as a caregiver?
- **For parents with children diagnosed with a condition through non-NBS means**
- How did you feel as you were trying to achieve a diagnosis for your child?
- How and who supported you in this time (prior to achieving a diagnosis)?
  - How did you feel when you received diagnostic confirmation of your child’s condition?
  - How were you supported at the time that the diagnosis was given?
  - What kind of information did you receive before your child had diagnostic testing?
  - Did you or your family experience any benefits or harms from the way that your child was diagnosed with their condition?
  - When you think back about the way your child was diagnosed, is there anything that could have been managed differently?
  - Has the timing of your child’s diagnosis changed the way you and your family manage your child’s condition in any way? (*prompts*: financial, health related, access to social support, psychological, access to research, reproductive planning)
  - How do you think you would have felt if you had have received your child’s genetic screening results earlier, while they were a newborn?
  - Have you participated in NBS for yourself or your children?
  - How useful do you feel NBS is? (*prompt* what are the potential benefits and harms)
  - If you opted out of NBS, can you tell me how and why you decided to opt out?
- **If your child was diagnosed through NBS, tell me about your experience of this pathway**
  - What type of information did you receive before you consented to NBS for your child?
  - Could the (method, type and timing) of information provided have been improved in any way before you consented for NBS?
  - How did you feel when you received a screen positive result for your family?
  - How were you supported at this time?
  - How did you feel when you received diagnostic confirmation of your child’s condition?
  - How were you supported at this time?
  - For parents/carers of children with a false positive screen (newborn screened positive for a named condition but was not confirmed to have this condition on diagnostic testing:) How did you feel when your child was not diagnosed with the screened condition? Did it affect your perspectives towards NBS as a whole? Would you go through the (NBS) process again if you were to extend your family? How could you have been better supported through this process?
  - For parents/carers of children with a false negative screen (newborn screened negative for a named condition but developed signs and symptoms and was later confirmed to have this condition on diagnostic testing: How did you feel when your child was diagnosed for a condition that may have been identified through NBS? Did it affect your perspectives towards NBS as a whole? Would you go through the (NBS) process again if you were to extend your family? How could you have been better supported through this process?
  - Tell me about the benefits of your child being diagnosed through NBS?
  - Did you or your family experience any harms from the way that your child was diagnosed with their condition?
  - When you think back about the way your child was diagnosed, is there anything that could have been managed differently?
  - What kind of support did you receive through the (screening, diagnostic and follow-up pathway). Would you have changed the way this support was given?
  - Looking back at your experiences, would you go through NBS again?
  - How valuable do you think NBS is?
  - Why did you agree to participate in NBS?
  - Would you change the NBS process in any way?
  - If your child’s results were uncertain (including false positives and false negatives), did this change your perspective of NBS?
  - How were you supported through the uncertainties, and could this have been managed differently?
  - Has the timing of your child’s diagnosis changed the way you and your family manage your child’s condition in any way? (*prompts*: financial, health related, access to social support, psychological, access to research, reproductive planning)

**Now lets’ talk about genomic NBS, where conditions are screened for on the dried blood spot using genomic sequencing technology (testing many or all genes at once).**

- - Tell me about your understanding of genomic NBS
  - What are your current perceptions of genomic NBS? (*prompt* is it useful, feasible)
  - What are the potential benefits of receiving a genetic diagnosis through NBS in the newborn period?
  - What are the potential risks of receiving a genetic diagnosis through NBS in the newborn period?
  - How do you think we can reduce the harms to families of receiving a genetic diagnosis through NBS?

**Model of care**

- In your perception, are there any conditions or group of conditions that definitely should be screened for through genomic NBS?
- In your perception, are there any conditions or group of conditions that definitely should not be screened for through genomic NBS?
- Do you think we should screen for individuals who are carriers of a genetic condition (and not just those who are at risk of being affected)?
- How much information do you think you need before participating in genomic NBS?
- Is the information you need to decide about participating in NBS different because the tests are genetic?
- How and when do you think this information should be delivered?
- How can we better support families through the genomic NBS pathway?
- Do you think you would like to access all the newborn screening results during the newborn period?
- Is there any other model of result disclosure that would be preferable to you? (*prompt* unlocking data over time or consenting for routine NBS and then separate consent for genomic NBS).
- How would you feel if the implications of the genetic change on NBS were uncertain? (*prompt* including difficulties in predicting a disease will occur in your child’s lifetime, difficulties in predicting the severity of a disease, difficulties in predicting a disease that will start in the newborn, infancy or early childhood period)
- How could we better support families in this uncertainty?
- How would you feel about genomic NBS for conditions that have an adult onset?
- How would you feel about genomic NBS for conditions that have no approved treatments currently?
- How would you feel about including conditions that just give you a risk of having a disease but no certainty of developing the disease?
- Do you have any feelings around targeting diseases for genomic NBS vs looking at the whole of your child’s genetic data through genomic sequencing?
- How would you feel about genomic NBS for conditions that occur primarily in selected ethnic groups?
- How do you think unexpected implications of results should be managed as part of genomic NBS?
- What are your perceptions about providing results of genetic testing for an infant who may feel differently about having this diagnosis as an adult? (*prompt* i.e. right of self determination of the child, concepts around autonomy of data)
- What do you feel about extending the role of NBS from benefiting the child alone to benefits for the wider family?
- How can we make sure that genomic NBS is useful and acceptable for all Australians?
- What are your views on insurance companies having access to genomic NBS data?
- Do you think adding genomic NBS will change the acceptability of NBS as a public health program?

**gEnomics4newborns: In-depth interviews with consumers, health professionals, scientists, and policy makers**

**Interview Discussion Guide – Health Professional/Scientist**

**General information:**

1. Introduce yourself and the purpose of the research. Newborn bloodspot screening (NBS) is a way of screening to detect individuals for whom early diagnosis and treatment of ‘actionable’ conditions offers health benefits primarily to the individual. Currently, Australia screens for around 50 conditions and there is over 99% public utilisation of the program. There is now potential to add genetic screening tests (either single gene tests, panels of genetic tests, using genomic technologies) for the first time to NBS, potentially allowing us to diagnose many more genetic conditions in the newborn period. However, there are potential benefits and harms, enablers and barriers to implementation of this new technology and we would like to explore these ideas with you as you are involved in screening, diagnosing and/or managing children with genetic diseases. The purpose of this research is to explore health care professional and scientists’ understanding of genomic NBS, your perceptions on its impact, potential benefits, harms and how the model of care should be developed. We will use this information to help us understand what is important for healthcare professionals and scientists in genomic NBS and how we may improve the health benefits, reduce the risks of harms to families, and provide a model which is equitable, fair, and ethical for all Australian families.
2. Are you happy to have the conversation recorded?
3. There are no right or wrong answers, feel free to provide as much or as little information as you like.
4. If you feel like you need a break, please feel free to stop at any time.
5. Even though we are recording our conversation, nothing you say will be linked back to you personally. The information we gather today will be combined with other interviews and reported as being anonymous.

- **Tell me about your career to date.**
  - How long have you been working in your current position?
  - How long have you been working in this field?
  - What does your work involve?
- **Tell me about your experiences of newborn screening or diagnosing children with genetic diseases?**
  - What are your clinical experiences with newborn screening?
  - What are your clinical experiences with diagnosing children with genetic diseases?
  - How often do you engage with parents and families in your role?
  - From your perspective, what is the difference to the (newborn, family and health system) in diagnosing children with clinical symptoms compared to receiving a newborn diagnosis through (newborn) screening?
  - Have you had any experience with genomic NBS?
  - What are the key supports provided in your setting for families receiving a genetic diagnosis for their child either through NBS programs or through clinical referral?
- **What is your experience with genomic sequencing technologies?**
- Can you describe your experiences.
- What is your experience of obtaining consent for NBS or genomic testing?
- **Now let us talk about genomic NBS in particular.**
- What are your perspectives on incorporating genomic sequencing technologies into NBS programs?
- Should genomic NBS from your perspective be in addition to or replace conventional tandem mass spectrometry?
- From your perspective what are the potential benefits of genomic NBS (*prompt* to the child, family, health system, society)
- From your perspective what are the potential harms of genomic NBS (*prompt* to the child, family, health system, society).
- Can you think of ways that that these potential harms may be mitigated?
- How do you think parents’ should consent to genomic NBS (opt in model, opt out model, additional consent on top of routine NBS panel)
- How much and what type of information should parents have before they decide to go through genomic NBS? (*prompt* Who should deliver this information?)
- What are the conditions or attributes of the conditions that should be part of genomic NBS (*prompt* incomplete penetrance, variable expression)
- What are the conditions or attributes of the conditions that should *not* be part of genomic NBS? (*prompt* adult-onset conditions, those with variable onset, those with variable severity, those that give you a risk of developing a disease, those where treatment options are limited).
- How and who should guide variant selection in terms of reporting genomic NBS results
- From your perspective, how should variants of uncertain significance be managed?
- From your perspective, how should unexpected findings be managed?
- How do we support families when they have uncertain, incidental, or false positive/false negative findings?
- Are there particular genetic results that should *not* be part of NBS reporting (*prompt*: carrier status, genetic results that show ‘risk’ of a disease, X-linked variants in females).
- How do you think we can preserve the high public utilisation of NBS programs if we are to incorporate genomics into NBS?
- Who do you think should have access to genetic data generated by genomic NBS?
- Where and how should this data be stored
- Are there any ethical implications of genomic NBS (*prompt*: rights to self determination of the child, autonomy)
- **Now thinking about equity in genomic newborn screening:**
- What comes to mind when you think about equity in genomic NBS?
- For rare diseases, there may be limited evidence on disease incidence and natural history, which may create inequities. What are your views on this?
  - Prompt: In what ways could this uncertainty be dealt with?
- What do you think needs to happen in Australia for genomic NBS to become integrated equitably into healthcare?
- **Policy and implementation of genomic NBS**
- Thinking about policies and frameworks that support implementation of genomic NBS, what are your perspectives on the current NBS National Policy frameworks that inform which conditions should be placed on NBS panels in Australia?
- Do you think these frameworks should also be used to consider which conditions are added/removed through genomic NBS?
- What do you think are the enablers of implementing genomic NBS?
- What do you think are the barriers to implementing genomic NBS?
- How do you think these barriers can be overcome?
- How do we ensure that genomic NBS is feasible and sustainable into the future?
- **Is there anything else you would like to discuss in terms of the equity, ethical or legal aspects of genomic NBS?**

**gEnomics4newborns: In-depth interviews with consumers, health professionals, scientists, and policy makers**

**Interview Discussion Guide – Policy maker^[[1]](#footnote-2)^**

**Introduction**

**Researcher introduction:** Name, role, organisation

I’m here to speak with you today about your views on the policy and implementation aspects regarding possible broader use of genomics in Australian newborn screening programs. We expect the interview will take approximately 45-60 minutes to complete.

Currently, Australia screens for around 50 conditions, primarily through biochemical testing methods, and there is more than 99% uptake of the programs suggesting high levels of public trust. There is the opportunity to further add genetic screening tests (either single gene tests, gene panels, or the use of genomic sequencing technologies) to newborn screening, potentially allowing us to identify hundreds of additional conditions in the newborn period. Along with the expected benefits of earlier detection of these conditions, there are potential harms, and there will be enablers and barriers to implementation of this new technology. We would like to explore these ideas with you as someone who is involved in newborn screening policy and/or implementation.

This study is being conducted by the University of Sydney, who will use the information to understand what is important for policy makers in the use of genomics in newborn screening and will inform the development of tools which will aim to streamline future government assessments of the use of genomic testing in newborn screening.

We want to emphasise that the information you provide will be kept secure and will not be passed onto any other person or organisation. This information will be combined with information from other participants, and you will not be named or identifiable in anyway.

As we noted in the consent form that you signed earlier, this study is voluntary, and you can choose to leave the discussion at any time. With your permission we would like to record the meeting. Is that ok with you? Do you have any questions about the interview before we start?

**Participant demographics**

We have a few questions about you and your role before we move onto the questions:

1. Name:
2. Organisation:
3. Role within organisation:
4. Date of interview:
5. Place of interview:

**Demographic questions**

1. How long have you worked in your current position?
2. How long have you worked in this field?
3. What has been your involvement with newborn screening policy or implementation? (*Prompts:* *involvement with HGSA; member of the PMC; involvement with the development of the National NBS Policy Framework*).
4. Do you have any clinical and/or laboratory experience relevant to this field?
5. What is your experience with population screening policy in general? *(Prompts: general experience in public health or public health ethics)*
6. What is your experience with the methods or government processes used for evaluating new health technologies such as genomic tests? (*Prompts*: *specific experience in HTA, or general experience in translating clinical and/or economic evidence into policy)*

**Newborn bloodspot screening and the potential use of genomics**

12. What are your views on the use of genomics in population screening in general or in newborn bloodspot screening in particular?

13. Is the use of genomics in newborn bloodspot screening being discussed or implemented in your organisation?

- How?
- Are the views of others in your organisation/field largely positive or negative towards the use of genomics in newborn screening?

14. What do you see as the biggest challenges for government decision makers around the use of genomics in newborn bloodspot screening?

15. How do you think incorporating genomics might impact current newborn bloodspot screening programs?

- What benefits do you see?
- Do you see any challenges?

**Policy regarding the use of genomics in newborn screening**

16. Who/which stakeholders do you think need to be engaged to inform policy regarding the use of genomics in newborn bloodspot screening?

17. What kinds of information do you think should be considered when making decisions about the use of genomics in newborn bloodspot screening?

- How do you think we should assess different genomic tests?
- Are there specific ethical or legal issues that should be considered?
- Are there specific social or equity issues that should be considered?

18. What are your views on the current Newborn Bloodspot Screening National Policy Framework?

- Do you think the current framework can be used to consider which conditions are added/removed if genomics is included as a screening test? *(Why/Why not?)*
- If you think the Framework needs to be changed to incorporate genomics, what process should be used to change the Framework, and who should be involved?

19. What are your views on the current process being used by government to expand the number of conditions screened in Australia? (*Prompts*: *get them to discuss the process itself, the stakeholders involved, and the information being relied on)*

- Do you think the current process is appropriate for considering the use of genomics in newborn bloodspot screening? *(Why/Why not?)*
- If you think the process needs to be changed to consider the use of genomics, what do you think would be the most important features of a new or modified process?

20. Under what circumstances do you think genomics should be included in newborn screening? (*Prompts: types of conditions (eg, monogenic/high penetrance conditions, conditions where there is at least one treatment available, national agreement on the addition*)

- Under what circumstances do you think genomics should **not** be included in newborn screening?

**Implementation of genomics in newborn screening**

21. What do you think are the challenges for implementing genomics in Australian newborn bloodspot screening programs?

- Overall program design?
- Harmonisation of programs across jurisdictions?
- What kinds of infrastructure are needed in health systems to implement and support genomics in newborn screening?
- What are the workforce implications?

22. Do you have any views on how these challenges could be addressed?

23. If genomics is more widely used in newborn bloodspot screening, what do you think would be the implications **for parents and families** for/of:

- Obtaining parents’ informed consent
- Receiving uncertain results
- Expectations regarding access to treatment and care
- Data security and privacy

**Suggestions**

24. What do you think would make the use of genomics in newborn bloodspot screening more or less acceptable for government decision makers?

25. Is there anything else you’d like to raise that we haven’t discussed?

**Snowball sampling:**

As part of recruiting people for this study, we’re asking participants if they know of any other suitable candidates. Are there any people that you think would be suitable for us to interview for this study? Are you happy to provide their name and I will contact them using publicly available information?

1. Guide informed by:

   Smith, H. S., Sherman, M., & Cardeiro, D. (2023). Conversations With the Editors: Stewardship in Genomic Medicine-Insights From Health Care Payers at the Forefront of Clinical Innovation and Partnerships. Clinical Therapeutics. <https://doi.org/10.1016/j.clinthera.2023.07.012>

   Cao, M., Notini, L., Ayres, S., & Vears, D. F. (2023). Australian healthcare professionals’ perspectives on the ethical and practical issues associated with genomic newborn screening. Journal of Genetic Counseling, 32(2), 376–386. https://doi.org/10.1002/jgc4.1645 [↑](#footnote-ref-2)
