# Supplementary material for: ‘Integrating Ethics and Equity with Economics and Effectiveness for newborn screening in the genomic age: A qualitative study protocol of stakeholder perspectives
Source: PLoS One. 2024 Mar 25;19(3):e0299336. doi: 10.1371/journal.pone.0299336 (PMC10962853; doi:10.1371/journal.pone.0299336)
Supplement: S2 Appendix — (DOCX) [file pone.0299336.s002.docx]

**S2 Appendix**

**gEnomics4newborns in-depth interviews with consumers, health professionals, scientists, and policy makers**

**RECRUITMENT SCREENER – PARENTS/CARERS (Cohorts 1a and 1b)**

**Your child’s diagnosis of a genetic condition**

**How was your child diagnosed with a genetic condition (tick the answer that most applies to you)**

My child was diagnosed through clinical referral (after having signs and symptoms of the genetic condition) □

My child was diagnosed due to a family history of a condition □

My child was diagnosed through a newborn screening program and received a true screen positive result (i.e. my child was diagnosed with the condition that was picked up through newborn screening) □

My child was diagnosed through a newborn screening program and received a false positive screening result (i.e. my child screened positive through newborn screening but did not end up being diagnosed with the condition) □

My child was diagnosed through a newborn screening program and received a false negative screening result (i.e. my child was diagnosed with a condition that could have been detected through newborn screening ) □

My child was diagnosed through prenatal (during pregnancy) screening □

Other (please specify) □ ­­­­­

**About you as a parent/carer**

Please state what applies to you as a parent/carer of a child with a genetic condition

**1. Gender**

- Female
- Male
- Non-binary / other
- Prefer not to say

**2. Age**

- 18-24
- 25-29
- 30-34
- 35-39
- 40-44
- 45-49
- 50-54
- 55-59
- 60-64
- 65-69
- 70-74
- 75+

**3. Postcode of residence**

*Free text*

**4. Aboriginal and Torres Strait Islander origin**

- No
- Yes, Aboriginal
- Yes, Torres Strait Islander
- Yes, Aboriginal and Torres Strait Islander
- Don’t know
- Prefer not to say

**5. Ancestry**

- Country of birth and/or language other than English spoken at home: (free text)

**6. Highest level of education completed (as per** [**ABS categories**](https://www.abs.gov.au/ausstats/abs@.nsf/Lookup/by%20Subject/4363.0~2017-18~Main%20Features~Education~53)**)**

***Highest year of school completed***

- Year 12 or equivalent
- Year 11 or equivalent
- Year 10 or equivalent
- Year 9 or equivalent
- Year 8 or below
- Never attended school.

***Highest non-school educational attainment***

- Postgraduate Degree
- Graduate Diploma / Graduate Certificate
- Bachelor Degree
- Advanced Diploma / Diploma
- Certificate III/IV
- Certificate I/II
- Certificate not further defined
- No non-school qualification
- Level not determined.

**7. Decision satisfaction/regret** [1]

**For participants whose child/ren received a genetic diagnosis within and outside of newborn screening:** Please reflect on the decision you made to participate in **further genetic testing after newborn screening.** By newborn screening we mean the heel prick test your baby received in hospital in the first few days after they were born to collect blood on a paper card to test for a range of conditions. Please show how strongly you agree or disagree with these statements by circling a number from 1 (strongly agree) to 5 (strongly disagree) which best fits your views about your decision.

**For participants of newborn screening whose child/ren received a false positive or false negative screen result:** Please reflect on the decision you made **to participate in newborn screening**. By newborn screening we mean the heel prick test your baby received in hospital in the first few days after they were born to collect blood on a paper card to test for a range of conditions. Please show how strongly you agree or disagree with these statements by circling a number from 1 (strongly agree) to 5 (strongly disagree) which best fits your views about your decision.

| It was the right decision | 1  Strongly agree | 2  Agree | 3  Neither agree nor disagree | 4  Disagree | 5  Strongly disagree |
| --- | --- | --- | --- | --- | --- |
| I regret the choice that was made | 1  Strongly agree | 2  Agree | 3  Neither agree nor disagree | 4  Disagree | 5  Strongly disagree |
| I would go for the same choice if I had to do it over again | 1  Strongly agree | 2  Agree | 3  Neither agree nor disagree | 4  Disagree | 5  Strongly disagree |
| The choice did me a lot of harm | 1  Strongly agree | 2  Agree | 3  Neither agree nor disagree | 4  Disagree | 5  Strongly disagree |
| The decision was a wise one | 1  Strongly agree | 2  Agree | 3  Neither agree nor disagree | 4  Disagree | 5  Strongly disagree |

**REFERENCES**

[1] Brehaut JC, O’Connor A, Wood T, et al. Validation of a Decision Regret Scale. Medical Decision Making 2003; 281–292.
