# Supplementary material for: ‘Integrating Ethics and Equity with Economics and Effectiveness for newborn screening in the genomic age: A qualitative study protocol of stakeholder perspectives
Source: PLoS One. 2024 Mar 25;19(3):e0299336. doi: 10.1371/journal.pone.0299336 (PMC10962853; doi:10.1371/journal.pone.0299336)
Supplement: Peer review history

# Editor report: Decision Letter 0

**DOI:** 10.1371/journal.pone.0299336.r001

**Authors:** Johanna Pruller

28 Feb 2024

‘Integrating Ethics and Equity with Economics and Effectiveness for newborn screening in the genomic age: a qualitative study protocol of stakeholder perspectives.

PONE-D-24-05175

Dear Dr. kariyawasam,

We’re pleased to inform you that your manuscript has been judged scientifically suitable for publication and will be formally accepted for publication once it meets all outstanding technical requirements.

Within one week, you’ll receive an e-mail detailing the required amendments. When these have been addressed, you’ll receive a formal acceptance letter and your manuscript will be scheduled for publication.

An invoice for payment will follow shortly after the formal acceptance. To ensure an efficient process, please log into Editorial Manager at http://www.editorialmanager.com/pone/, click the 'Update My Information' link at the top of the page, and double check that your user information is up-to-date. If you have any billing related questions, please contact our Author Billing department directly at authorbilling@plos.org.

If your institution or institutions have a press office, please notify them about your upcoming paper to help maximize its impact. If they’ll be preparing press materials, please inform our press team as soon as possible -- no later than 48 hours after receiving the formal acceptance. Your manuscript will remain under strict press embargo until 2 pm Eastern Time on the date of publication. For more information, please contact onepress@plos.org.

Kind regards,

Johanna Pruller, Ph.D.

Associate Editor

PLOS ONE

Journal Requirements:

1. Thank you for stating the following financial disclosure: 

"SN, SK, JS, AJN, SC, MF, MO. SK, JS, CM, KB, JW, NM

This study was funded by The Commonwealth of Australia Medical Research Future Fund, 2021 Genomics Health Futures Mission under the activity title gEnomics4newborns: integrating Ethics and Equity with effectiveness and economics for genomic newborn screening (APP2015965)."

Please state what role the funders took in the study.  If the funders had no role, please state: "The funders had no role in study design, data collection and analysis, decision to publish, or preparation of the manuscript." 

If this statement is not correct you must amend it as needed. 

 

Please respond by return e-mail so that we can amend your financial disclosure and competing interests on your behalf.

2. Thank you for stating the following in the Competing Interests section: 

"I have read the journal's policy and the authors of this manuscript have the following competing interests: AJN is a member of the New South Wales Ministry of Health Newborn Screening Expert Advisory Group. All other authors declare no competing interests."

Please confirm that this does not alter your adherence to all PLOS ONE policies on sharing data and materials, by including the following statement: "This does not alter our adherence to  PLOS ONE policies on sharing data and materials.” (as detailed online in our guide for authors http://journals.plos.org/plosone/s/competing-interests). If there are restrictions on sharing of data and/or materials, please state these. Please note that we cannot proceed with consideration of your article until this information has been declared. 

 

Please respond by return email with your amended Competing Interests Statement and we will change the online submission form on your behalf.

3. Please provide a complete Data Availability Statement in the submission form, ensuring you include all necessary access information or a reason for why you are unable to make your data freely accessible. If your research concerns only data provided within your submission, please write "All data are in the manuscript and/or supporting information files" as your Data Availability Statement.

# Editor report: Acceptance letter

**DOI:** 10.1371/journal.pone.0299336.r002

**Authors:** Johanna Pruller

12 Mar 2024

PONE-D-24-05175 

PLOS ONE

Dear Dr. kariyawasam, 

I'm pleased to inform you that your manuscript has been deemed suitable for publication in PLOS ONE. Congratulations! Your manuscript is now being handed over to our production team.

At this stage, our production department will prepare your paper for publication. This includes ensuring the following:

* All references, tables, and figures are properly cited

* All relevant supporting information is included in the manuscript submission,

* There are no issues that prevent the paper from being properly typeset

If revisions are needed, the production department will contact you directly to resolve them. If no revisions are needed, you will receive an email when the publication date has been set. At this time, we do not offer pre-publication proofs to authors during production of the accepted work. Please keep in mind that we are working through a large volume of accepted articles, so please give us a few weeks to review your paper and let you know the next and final steps. 

Lastly, if your institution or institutions have a press office, please let them know about your upcoming paper now to help maximize its impact. If they'll be preparing press materials, please inform our press team within the next 48 hours. Your manuscript will remain under strict press embargo until 2 pm Eastern Time on the date of publication. For more information, please contact onepress@plos.org.

If we can help with anything else, please email us at customercare@plos.org.

Thank you for submitting your work to PLOS ONE and supporting open access. 

Kind regards, 

PLOS ONE Editorial Office Staff

on behalf of

Dr. Johanna Pruller 

Staff Editor

PLOS ONE
